# Knowledge, attitude and perception of West Africans towards COVID-19: a survey to inform public health intervention

**DOI:** 10.1186/s12889-022-12814-9

**Published:** 2022-03-05

**Authors:** Aniefiok John Udoakang, Alexandra Lindsey Djomkam Zune, Kesego Tapela, Oloche Owoicho, Ifeoluwa Kayode Fagbohun, Claudia Adzo Anyigba, Mat Lowe, Nora Nghochuzie Nganyewo, Bineta Keneme, Frances Ngozi Olisaka, Agatha Nkem Henry-Ajala, Mary Aigbiremo Oboh, Lily Paemka, Lucas N Amenga-Etego

**Affiliations:** 1 West African Centre for Cell Biology of Infectious Pathogens (WACCBIP), College of Basic and Applied Sciences, University of Ghana, Legon, Accra Ghana; 2 Department of Biological Sciences, University of Medical Sciences, Laje Road, P.M.B. 536, Ondo City, Ondo State Nigeria; 3 Department of Biochemistry, Cell and Molecular Biology, College of Basic and Applied Sciences, University of Ghana, Accra, Ghana; 4 Department of Biological Sciences, Benue State University, Makurdi, Nigeria; 5 Public Health Division, Nigeria Institute of Medical Research, Lagos, Nigeria; 6 Society for the Study of Women´s Health (SSWH), Old Yundum, Gambia; 7 Medical Research Council Unit, London School of Hygiene and Tropical Medicine, Banjul, Gambia; 8 Département de Biologie Animale, Faculté des Sciences et Techniques, Equipe Génétique et Gestion pour les Populations, Université Cheikh Anta Diop de Dakar, BP. Box 5005, Dakar, Sénégal; 9 Environmental and Public Health Microbiology, Department of Biological science, Benson Idahosa University, P.M.B 1100, Benin City, Edo State Nigeria; 10 Department of Zoology, Parasitology & Bioinformatics Unit, University of Lagos, Lagos, Nigeria

**Keywords:** COVID-19, West Africa, Knowledge, attitude and perception, Vaccine acceptance

## Abstract

**Background:**

The first case of the novel coronavirus disease-2019 (COVID-19) in West Africa was first confirmed in Nigeria in February 2020. Since then, several public health interventions and preventive measures have been implemented to curtail transmission of the causative agent, the Severe Acute Respiratory Syndrome Coronavirus 2 (SARS-CoV-2). Therefore, this study was performed to assess the knowledge, attitudes, and perceptions of West Africans towards COVID-19.

**Methods:**

An online survey was conducted between 29 September to 29 October 2020 among West Africans. Thirty-three survey questions were designed to collect sociodemographic data and participants’ knowledge, attitude and perception towards COVID-19. The study targeted all West African nationals who were 18 years and above, and willing to participate in the study. Participants were either in-country or abroad.

**Results:**

Overall, 1106 respondents (≥18 years) from 16 West African countries, with about 12.1% of them residing outside the West African subregion, participated in the survey. The respondents had an average COVID-19 knowledge score of 67.82 ± 8.31, with knowledge of the disease significantly associated with the country of residence (*p* = 0.00) and marginally (*p* = 0.05) so with settlement types (i.e., urban, suburban and rural areas). Most respondents (93.4%) could identify the main COVID-19 symptoms, and 73.20% would consult a healthcare professional if infected with SARS-CoV-2. Also, 75.2% of the respondents are willing to receive the COVID-19 vaccine, whereas 10.40% and 14.40% are unwilling and undecided, respectively. Perceptions of what constitute COVID-19 preventive measures were highly variable. Approximately, 8% of the respondents felt that their government responded excellently in managing the pandemic while a third felt that the response was just good. Also, more than half (54%) opined that isolation and treatment of COVID-19 patients is a way of curbing SARS-CoV-2 spread.

**Conclusions:**

Most West Africans have basic knowledge of COVID-19 and showed a positive attitude, with likely proactive practice towards the disease. However, results showed that these varied across countries and are influenced by the types of settlements. Therefore, the health and education authorities in various countries should develop focused measures capturing people in different settlements to improve their preventative measures when designing public health interventions for COVID-19 and any future epidemics or pandemics.

**Supplementary Information:**

The online version contains supplementary material available at 10.1186/s12889-022-12814-9.

## Introduction

Since the declaration of the novel coronavirus disease-2019 (COVID-19) as a pandemic by the World Health Organization on 11 March 2020, its global burden keeps rising daily [1]. The virus has infected about 199 million people with more than 4.2 million deaths reported worldwide (as of 3 August 2021) [2].

In West Africa, the first case of COVID-19 was confirmed in Nigeria on 27 February 2020 and within 1 month, the virus had spread to all 16 countries [3]. Public awareness plays a major role in preventing the spread of infectious diseases and outbreaks particularly in settings with poor infrastructure and healthcare systems, akin to middle and low-income countries, including those in West Africa, with limited capacity to cater for disease outbreaks [4].

Safety guidelines such as regularly sanitizing hands with hydroalcoholic solutions, washing hands with soap and water [5], wearing face masks [3], quarantining of suspected cases, isolation and social distancing, including travel restrictions and banning gatherings of more than ten people [6], have been adopted. Proper adherence to these control measures primarily stems from the publics’ knowledge, attitudes, and perception (KAP) of the disease [7]. This study investigated the current knowledge, attitudes and perceptions of the people of West African origin towards the COVID-19 pandemic. The findings therein will be geared towards informed policies and decisions to handle the spread of the virus and curtail the outbreak of the disease in the subregion.

## Methods

### Study setting

**Fig. 1:**
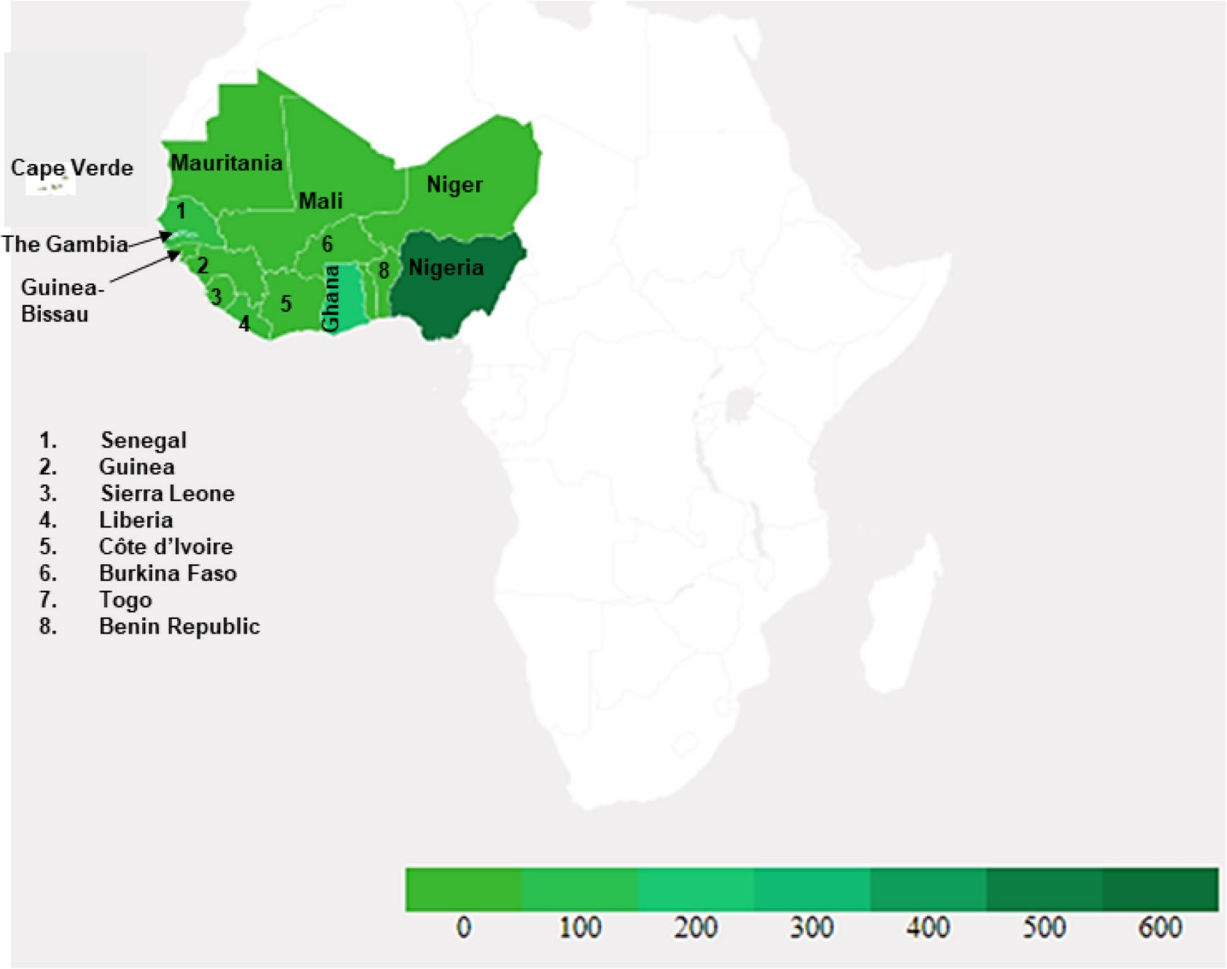
Country of Origin of Respondents. The shaded areas represent the 16 West African countries. The intensity of the green colour indicates the number of respondents from the respective countries

This study was conducted in West Africa comprising 16 countries (Fig. 1), which were all eligible for the study. These countries are distributed over an estimated 6,140,000 km^2^, approximately one-fifth of the African continent and lying between latitudes 4°N and 28°N and longitudes 15°E and 16°W [8]. These countries include Benin, Burkina Faso, Cape Verde, Côte d’Ivoire, The Gambia, Ghana, Guinea, Guinea-Bissau, Liberia, Mali, Mauritania, Niger, Nigeria, Senegal, Sierra Leone and Togo [9]. West African population is about 408 million, representing 5% of the world’s population with 56% under the age of 20 years and 47.7% living in urban areas [10]. Participants were considered for inclusion in the study if they were of West African origin, aged 18 years and above.

### Questionnaire design, piloting and validation

An electronic cross-sectional survey was designed to assess the general knowledge, attitude, and perception of West Africans towards the COVID-19 pandemic. The questionnaire was initially drafted in English by our research team and the survey content was critically reviewed. Then it was translated to French, by a professional translator considering there are nine Francophone West African countries. Subsequently, the questionnaire was randomly administered to selected contacts for piloting to assess comprehension, challenges, and minimum time needed in taking the survey. Those who responded to the questionnaire in the pilot phase were not members of the research team. Information gathered during this pre-testing phase was used to fine-tune the questionnaire accuracy for better comprehension and estimation of time required to complete the survey before making it available to the study participants online.

### Questionnaire’s content

The survey questions (31) were designed to obtain participants’ demographic characteristics and assess their knowledge of COVID-19, attitude, and perception (Supplementary mateCite ESM.rial: Survey questions). While some questions required selecting the right answer(s), others needed either a yes/no, true/false or I don’t know responses. For questions on attitude and perception, participants were required to respond by scoring on a five-point Likert scale (1, strongly disagree; 2, disagree; 3, neutral; 4, agree; 5, strongly agree). Hence, no question was left open-ended.

### Data collection procedure

A self-administered online questionnaire disseminated over different platforms, including Email, Telegram, Twitter, WhatsApp, Instagram, and Facebook, through a Google Doc URL link was used to collect data. Data from a similar study conducted to examine Gambian adults’ perceptions and behaviours in response to COVID-19 social mitigation strategies were also extracted with permission [11]. An email address, checked daily, was provided for participants to seek further information on the study if needed. Data were collected between 29 September and 29 October 2020.

### Data management and analysis

Data from the online survey were extracted into Microsoft Excel, cleaned and verified by members of the research team, including the statistician. Participants’ demographics and research variables were described using descriptive statistics. For the research variables, a correct answer was assigned one point, and an incorrect/unknown answer was assigned zero point. The total knowledge score ranged from zero to 29, which was computed and converted to percentages for each respondent. Participants’ general knowledge, using Bloom’s cut-off point, was classified as good [80% and 100%], moderate [60% and 79%], and poor [≤59%] knowledge. Bivariate analysis was performed using Pearson’s Chi-square test while Fisher’s exact test was carried out to assess the association between demographic variables and COVID-19 knowledge, attitude and perception. Ordinal logistic regression was used to investigate the factors affecting COVID-19 knowledge and perception. All significant variables (*p*-value < 0.05) from the bivariate analysis were incorporated into the multivariate analysis to estimate the multivariable model. Also, variables hypothesized to be associated with the level of knowledge and perception of COVID-19 were incorporated in the multivariate model. The statistical significance level was set at *p <* 0.05. The Statistical Package for the Social Sciences (SPSS) version 25 [12] was used to perform all analyses.

### Ethical consideration

 This study was approved by the Ethics Committee for Basic and Applied Sciences (ECBAS 063/19-20) at the University of Ghana and conformed to the Declaration of Helsinki ethics guidelines. The raw dataset was saved in a secure password-protected folder and the verified dataset was anonymized to ensure confidentiality. Participation was voluntary, and informed consent was documented on the first page of the survey. Participants who changed their minds in the course of taking the survey were free to opt-out of submitting the completed questionnaire and their initial responses were not included in the final analyses.

## Results

### Sociodemographic characteristics of the respondents

The sociodemographic characteristics of the respondents are presented in Table 1. A total of 1106 West African adults (≥18 years) residing within 975 (87.9%) and outside 131 (12.1%) the region, respectively, completed the online survey, with 50.4% females and 49.5% males. Responses were received from all the 16 eligible West African countries. Figure 1 shows the respondents country of origin - the intensity of the green colour corresponds with the number of respondents from each country. The average household size was 5.33 ± 3.853, with 52.5% of respondents having between four and six children. Describing their area of residence, most (75.9%) indicated that they lived in an urban setting. Age group composition includes ≤ 20 years (3.9%), 21 – 30 years (41.8%), 31 – 40 years (39.1%) and ≥ 41 years (12.2%), with a mean age of 32.14 ± 8.359 years, and missing age for 3% of the participants. More than half (51.7%) of the respondents were married, and 51.4% identified as Christians. Majority (90.6%) had tertiary education and 64.6% worked in the public or private sector, apart from health care professionals (11.4%). Table 1 shows the distribution of participants by country of origin with the highest number (49.6%) of respondents from Nigeria.

**Table 1:** Sociodemographic factors of the respondents

**Variables**		**Number of respondents (%)**	**Mean ± SE**
**Country of residence**	Nigeria	470 (42.5)	
	Ghana	184 (16.6)	
	The Gambia	179 (16.2)	
	Senegal	75 (6.8)	
	Liberia	28 (2.5)	
	Other WAC	39 (3.5)	
	Outside WA	131 (11.8)	
**Gender**	Male	547 (49.5)	
	Female	557 (50.5)	
**Age group**	≤ 20	43 (3.9)	
	21 – 30	462 (41.8)	
	31 – 40	432 (39.1)	
	≥ 41	135 (12.2)	
**Mean Age**		1071	32.14 ± 8.359
**Educational status**	Senior Secondary	92 (8.3)	
	Tertiary	1002 (90.6)	
	Vocational	9 (0.8)	
**Total years of formal education**		1070	17.28 ± 4.374
**Household size**	1–3	282(26.3)	
	4–6	563(52.5)	
	> 6	228(21.2)	
**Mean household size**		1073	5.33 ± 3.853
**Area description**	Urban	693 (75.9)	
	Rural	25 (2.6)	
	Suburban	196 (21.4)	
**Occupation**	Student	186 (16.8)	
	Public/Private servant	714 (64.6)	
	Unemployed	74 (6.7)	
	Health Professionals	126 (11.4)	
**Marital Status**	Single	499 (46.5)	
	Married	554 (51.7)	
	Others	19 (1.8)	
**Religious affiliation**	Christian	568 (80.3)	
	Islam	125 (17.7)	
	Others	12 (2.0)	

*WA* West African, *WAC* West African Countries, Religious affiliation (Others): Traditional religion and no response; Marital status (Others): Divorced, widowed and separated

### Knowledge of respondents about COVID-19: causative agent, means of transmission, main symptoms, high-risk group and places, and who should be tested

The respondents’ knowledge score on COVID-19 is presented in Table 2, with an average knowledge score of 67.82 ± 8.31. The knowledge score was significantly associated with the country of residence (*P* = 0.000) and marginally with settlement type (*p *= 0.05), but not with gender, age, occupation, religion, marital and educational status.

**Table 2:** Knowledge score of the respondents and multiple linear regression on factors associated with COVID-19 knowledge

**Variables**		**Knowledge score (%)**	**Coefficients ± SE**	***p*** **-value**
**Country of residence**	Nigeria	68.50 ± 8.23	-0.649 ± 0.137	0.000
	Ghana	68.33 ± 7.57		
	The Gambia	69.06 ± 8.68		
	Senegal	66.86 ± 8.39		
	Liberia	69.47 ± 8.86		
	Other WAC	62.03 ± 8.70		
	Outside WA	70.49 ± 7.75		
**Gender**	Male	67.88 ± 8.72	0.388 ± 0.635	0.32
	Female	68.44 ± 8.20		
**Age group**	≤ 20	68.37 ± 7.94	0.395 ± 0.319	0.86
	21 – 30	68.27 ± 8.89		
	31 – 40	67.90 ± 8.31		
	41 and above	68.63 ± 7.61		
**Area description**	Urban	69.58 ± 8.33	0.444 ± 0.373	0.05
	Rural	69.36 ± 8.11		
	Suburban	68.01 ± 8.11		
**Religious affiliation**	Christian	67.83 ± 9.61	1.010 ± 0.645	0.75
	Islam	69.77 ± 9.46		
	Others	29.29 ± 3.57		
**Educational status**	Senior Secondary	29.30 ± 3.63	2.104 ± 1.475	0.07
	Tertiary	33.50 ± 3.32		
	Vocational	29.67 ± 3.64		
**Occupation**	Student	29.19 ± 3.58	2.104 ± 1.475	0.06
	Public/Private servant	28.00 ± 4.63		
	Unemployed	29.70 ± 3.51		
	Health Professionals	68.31 ± 8.61		
**Marital Status**	Single	67.98 ± 8.31	-0.187 ± 0.660	0.63
	Married	69.65 ± 7.65		

*WA* West African, *WAC* West African Countries; Religion (Others): Traditional religion and no response; Marital status (Others): Divorced, widowed and separated

**Fig. 2:**
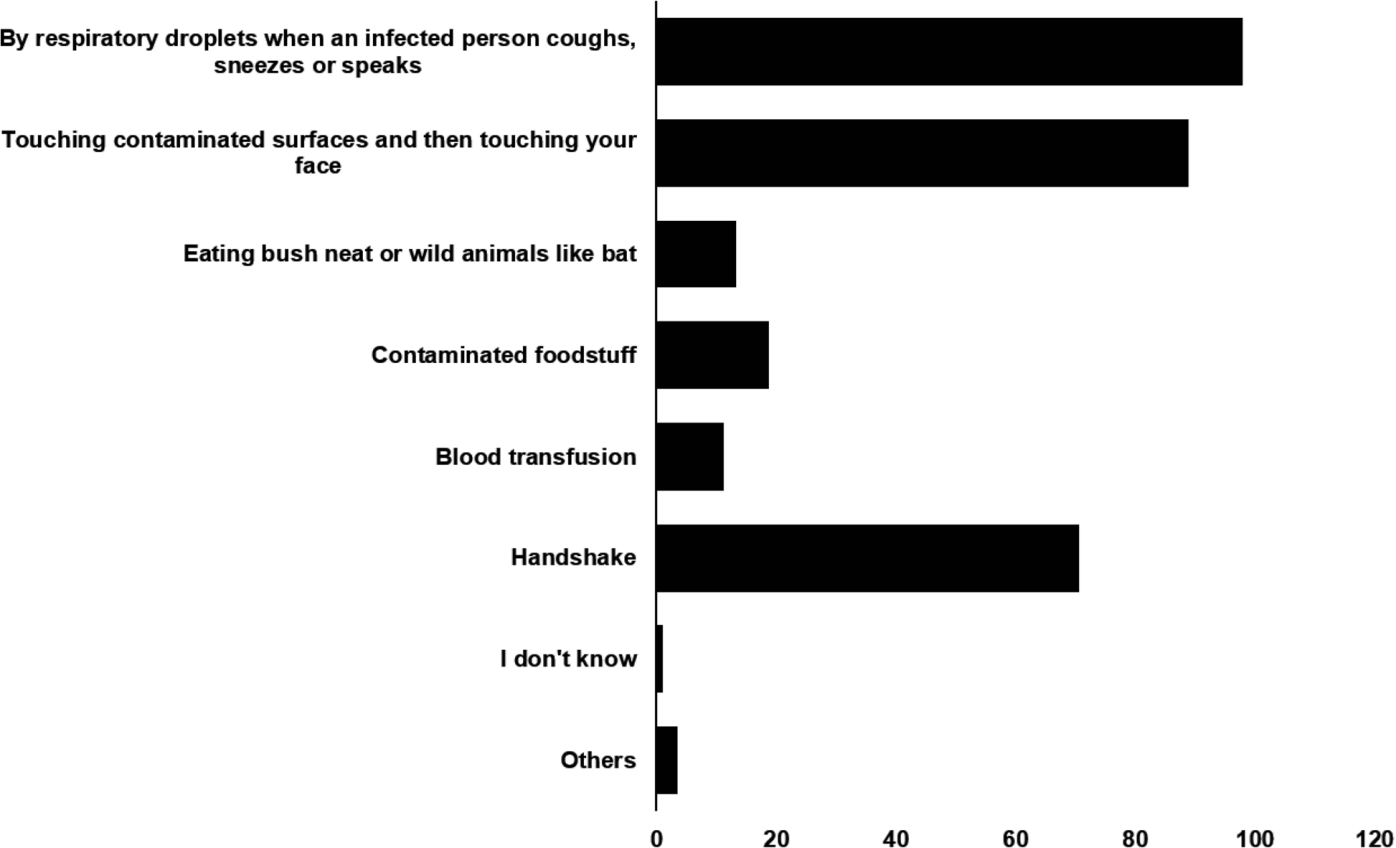
Participants response to “How is SARS-CoV-2 is transmitted?”

**Fig. 3:**
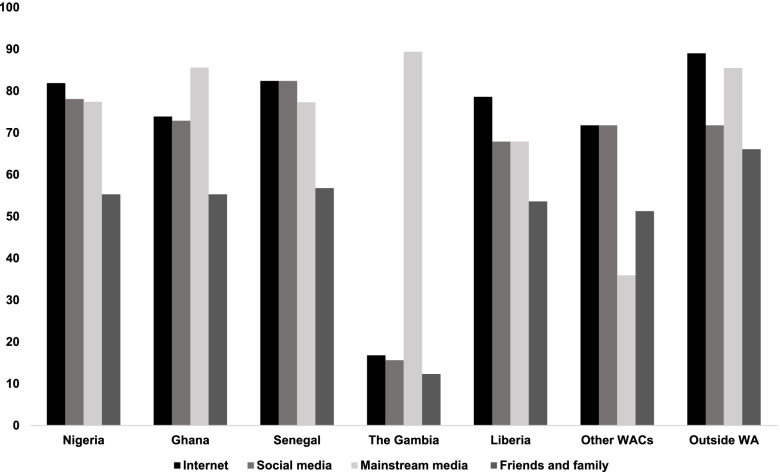
Participants sources of COVID-19 Information (Internet include: Journal articles, virtual conferences, online information; social media include: WhatsApp, Twitter, Instagram, Facebook, etc.; Mainstream media: Television, Radio and Print media)

Notably, on the question on “How is SARS-CoV-2 is transmitted?”, 80.80% of the respondents correctly agreed that the disease can be transmitted by respiratory droplets when an infected person coughs, sneezes or speaks. Additionally, 73.40% correctly agreed that SARS-CoV-2 can be contracted by touching contaminated surfaces and then touching one’s face (Fig. 2). Regarding clinical symptoms, most of the respondents (93.40%) agreed that chest pain, fever, dry cough, shortness of breath are the main symptoms of COVID-19. However, only 31.30% know that, unlike the common cold, stuffy nose, runny nose, and sneezing are less common in persons with COVID-19. The proportion of respondents who correctly identified the people most at risk of contracting COVID-19 is 75.1%: people who work in high-risk settings such as hospitals, prisons and other closed settings, 83.7%: overseas travellers, 71.6%: researchers whose research involves close contact with the causative virus or people with COVID-19 and 72.8%: people with compromised immune system. In addition, 62.8% correctly identified old age, underlying chronic diseases and obesity as predisposing factors for severe COVID-19 conditions. Most of the respondents (80.10%) acquired information about COVID-19 from the mainstream media while 66.70%, 64.70% and 45.5% acquired the information from the internet, social media and their friends and family members, respectively (Fig. 3).

### Attitude toward COVID-19: vaccine acceptance, worried about getting infected and what to do if infected with SARS-CoV-2

The willingness of the respondents to receive COVID-19 vaccine is presented in Table 3. Overall, 75.4% are willing to accept COVID-19 vaccine with the highest level of acceptance from those residing in Ghana (92.4%) and the lowest from The Gambian (38.1%) residents, who were mostly (41.5%) undecided regarding COVID-19 vaccine acceptance. The willingness to accept COVID-19 vaccine was significantly associated (*p* = 0.000) with country of residence, age category, religious affiliation, occupation, educational status (*p* = 0.013) and marital status (*p* = 0.026), but not gender (*p* = 0.89) and type of settlement (*p* = 0.95).

**Table 3:** Willingness to accept COVID 19 vaccine

Variables		Yes (75.1%)	No (9.9%)	Undecided 14.6 (%)	*p*-value
**Country of residence**	Nigeria	398 (84.7)	30 (6.4)	42 (8.9)	0.000
	Ghana	170 (92.4)	6 (3.3)	8 (4.3)	
	Senegal	40 (54.1)	18 (24.3)	16 (21.6)	
	Other WAC	29 (74.4)	5 (12.8)	5 (12.8)	
	Gambia	67 (38.1)	36 (20.5)	73 (41.5)	
	Liberia	16 (57.1)	8 (28.6)	4 (14.3)	
	Outside WA	110 (84.6)	6 (4.6)	14 (10.8)	
**Gender**	Male	414 (75.8)	55 (10.0)	77 (14.1)	0.89
	Female	414 (74.6)	59 (10.6)	82 (14.8)	
**Age group**	≤ 20	28 (66.7)	8 (19)	6 (14.3)	0.000
	21—30	318 (69)	61 (13.2)	82 (17.8)	
	31—40	352 (81.5)	37 (8.6)	43 (10)	
	41 and above	115 (85.2)	5 (3.7)	15 (11.1)	
**Area description**	Urban	574 (83.1)	57 (8.2)	60 (8.7)	0.95
	Rural	20 (80)	2 (8)	3 (12)	
	Suburban	163 (83.2)	18 (9.2)	15 (7.7)	
**Religious affiliation**	Christian	528 (93)	17 (3)	23 (4)	0.000*
	Islam	92 (74.8)	17 (13.8)	11.4 (11.4)	
	Others	11 (91.7)	-	1 (8.3)	
**Educational status**	Senior Secondary	57(62.6)	17(18.7)	17(18.7)	0.013
	Tertiary	764 (76.4)	95 (9.5)	141 (14.1)	
	Vocational	7 (77.8)	2 (22.2)	0	
**Occupation**	Student	133 (71.9)	31 (16.8)	21 (11.4)	0.000*
	Public/Private servant	553 (77.7)	60 (8.4)	99 (13.9)	
	Unemployed	31 (41.9)	16 (21.6)	27 (36.5)	
	Health Professionals	110 (87.3)	6 (4.8)	10 (7.9)	
**Marital Status**	Single	370 (74.4)	63 (12.7)	64 (12.9)	0.026
	Married	424 (76.5)	48 (8.7)	82 (14.8)	
	Others	19 (100)	-	-	
Total		828 (75.2)	114 (10.4)	159 (14.4)	

*WA* West African, *WAC* West African Countries; Religion (Others): Traditional religion or no response; Marital status (Others): Divorced, widowed or separated

**Fig. 4:**
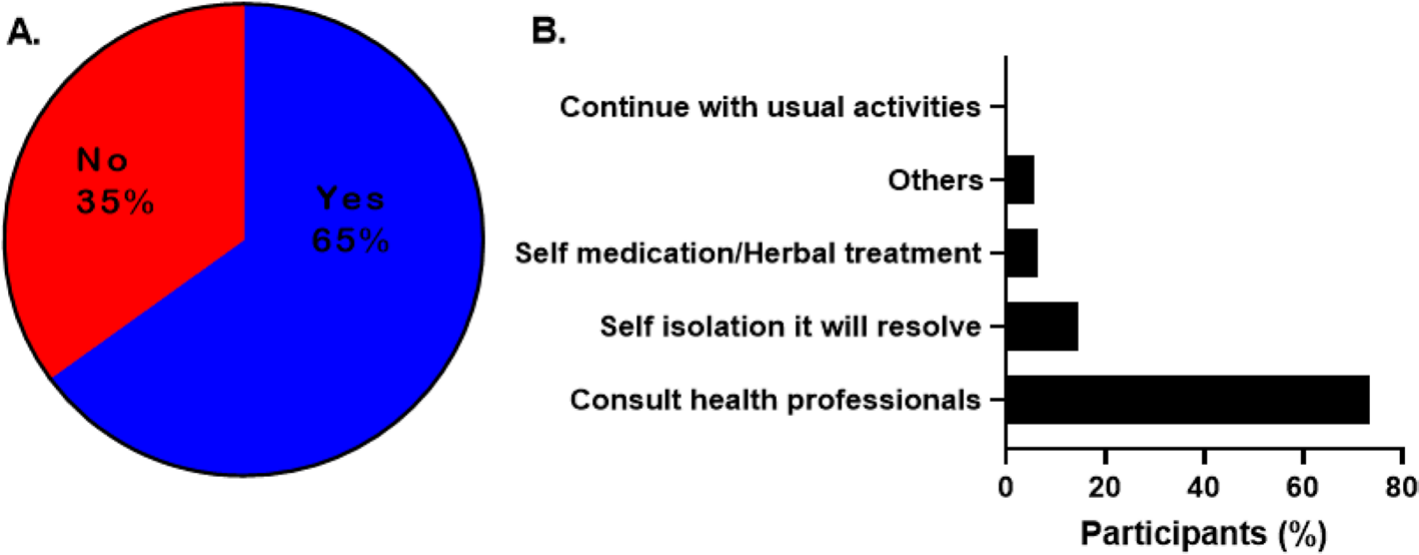
Attitude of the respondents toward COVID-19. **A** Worried about contracting SARS-CoV-2 **B** Response to “What will you do if you contract COVID-19?”

In addition, most of the respondents (65.30%) were worried that they or someone they know may contract COVID-19 (Fig. 4 A). Similarly, most of the respondents (73.20%) reported willingness to consult health care professionals while 14.50%, 6.40% and 5.60% will go on self-isolation, self-medication/herbal treatment, or other means of treatment, respectively, if they contract the disease (Fig. 4B).

### Perceptions on COVID-19: how to prevent getting infected and/or spreading the virus and perception of government intervention

**Fig. 5:**
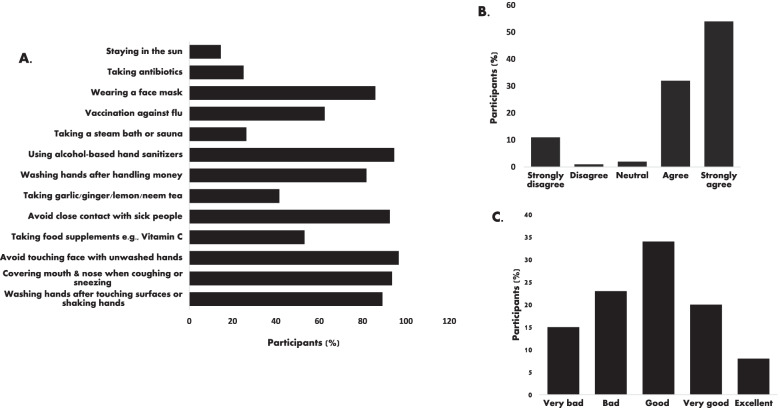
Perceptions on COVID-19 control and governments’ response **A** Preventive measures **B** Response to “Isolation and treatment of COVID-19 patients is best for the management of the COVID-19” **C** Government’s response to COVID-19 pandemic

Most of the respondents (96.7%) perceived that avoiding touching your eyes, nose, and mouth with unwashed hands helps prevent SARS-CoV-2 spread. Similarly, 94.6%, 93.6% and 92.6% respectively perceived that ‘the use of alcohol-based hand sanitisers, covering one’s mouth when coughing or sneezing and avoiding close contact with sick people’ could prevent contracting or spreading SARS-CoV-2 (Fig. 5 A). On the other hand, perceptions of what constitute COVID-19 preventive measures were highly variable, including getting a vaccination against flu (62.5%), taking food supplements e.g., Vitamin C (53.2%), eating garlic (41.6%), ginger or drinking lemon or neem tea (26.4%), steaming or taking a hot bath/sauna (25.1%), taking antibiotics (19.4%), gargling mouthwash and/or saline water and staying under the sun (14.6%) (Fig. 5 A). On treatment and management of COVID-19 patients, 86.1% agreed that isolation and treatment of COVID-19 patients are best for managing the disease (Fig. 5B). Additionally, 8% perceived that their governments’ response to the pandemic was “Excellent” while 20% and 34% stated the response was “Very good” and “Good”, respectively (Fig. 5 C) and more than half of the respondents felt that prompt measures were taken by their government to curb the SARS-CoV-2 spread.

## Discussion

Risk perception is key in defining disease prevention, control, treatment, eradication, or elimination [13]. Varied COVID-19 burdens and responses have been reported globally, partly attributable to the varied knowledge, attitude and perception of the disease, which generally define risk perception [14]. As a novel disease, people’s perception will probably border on the availability of quality and reliable data generated through research, public engagement and education, as well as the prevalence of the disease in different geographical locations. Despite the potential for under-reporting across Africa, the low case and mortality reports in official realms in Africa [15] underscores the need for this survey, which aims to ascertain the knowledge, attitude and perception of West Africans towards the disease. Generally, males are more inclined to fill out surveys than females [16]; however, there were one per cent more female participants in this survey than males [17]. Additionally, analysis of the results indicated that the majority of the respondents were either in the tertiary institution or had attained tertiary level education. By and large, the nature of the present survey could explain these findings; being an online survey, respondents were more likely to be educated. Thus, their respective participation might have been influenced by their basic understanding of the pandemic, ability to assimilate and interpret circulating information, and potential to form meaningful opinions [18].

From our survey, country of residence and type of settlement significantly correlated with knowledge of the pandemic. This significant variation could be attributed to the huge variation in the breadth of mainstream media (e.g. radio, TV) coverage and availability of locally generated and globally available information, local disease burden and ability to infer from available information [14, 19], which may explain the variation in the depth of content and ability to identify groups of people most at risk to COVID-19. This is the medium of information exchange, and this study shows the immense contribution of mainstream media as an outlet for information on the pandemic. However, a similar study in Central Africa showed relatively low average knowledge of COVID-19 in people living in the rural and suburban areas despite 74.7% of them having a university education [20]. In our study, more than three-quarter (75.9%) of the respondents resided in an urban setting, with majority either in higher institutions or having obtained a degree (average age of formal education = 17.28 ± 4.37), which may explain the high knowledge level observed in this study. This is not unlikely as more than half of the world’s population are urban dwellers [21], who have more access to services like communication, including cellular network, and education [22]. Also, people with a high educational qualification will be more drawn towards responding to an online survey. The majority of the respondents were between 21 and 30 years followed by those 31 – 40 years of age. This group of participants, under age 40, seem to be the most active on social media [23]; however, inferring from the results, mainstream media was the best information outlet, followed by social media in West Africa. This is despite the high prevalence of misinformation and conspiracy theories around COVID-19 within the social media ecosystem, owing to the fact that most households own a mobile phone in Africa [24] and social media is a powerful source of information. The disease prevalence can also inform people, as individuals with close contact with the disease, including frontline health workers, researchers, and others who have had the unfortunate experience of losing loved ones to COVID-19 or witnessed relatives with mild to severe COVID-19 symptoms will much more appreciate the disease severity. This may have influenced their knowledge of the disease, which may explain the knowledge scores observed, and the overall knowledge on the COVID-19 affects attitude towards the pandemic [23]. The knowledge scores observed may be attributable to the success of sensitization campaigns such as that by the West African Health Organization [25, 26].

The attitude of respondents towards COVID-19 was measured in many ways, including their fear of contracting the virus and their response to such an event. Generally, the majority of respondents dreaded the thought of they or any of their relations contracting the virus. The concern of contracting SARS-CoV-2 by most participants is a testament to their knowledge of consulting health professionals at the onset of COVID-19 symptoms [27]. Willingness to accept a COVID-19 vaccine was another means of ascertaining the attitude of respondents to the disease. In this study the willingness to accept a vaccine was generally good; however, of concern is the influence that those unwilling to accept the vaccine may have on those that are undecided, who unfortunately constituted more than the unwilling [28]. These uncertain individuals are not necessarily part of the anti-vaccine movement but may need more compelling information showing the need for a vaccine, that is individual and population-level benefits. Such individuals have vaccine safety issues or probably still think the virus’s existence is a hoax [28, 29]. Also, the overall attitude of respondents is significantly associated with age. Age reflects an awareness of the importance of understanding relevant information [17] and may explain the significant association between age and attitude towards the pandemic. In this study, it was observed that increasing age led to better attitude and positive response towards COVID-19 vaccine acceptance, as 85.2% of respondents, who were 41 years and above, were willing to be vaccinated compared to 66.7% of those 20 years and below. The attitude of respondents is associated significantly with religion. Religion is quite instrumental in knowledge acquisition, attitude and perception formation, much so in this pandemic [30]. Some individuals believe that the pandemic is a supernatural purge that will culminate in the end times. This may explain the significant association with religion. Conversely, existing religious inequalities have been exacerbated by COVID-19 and may affect efforts to curb the pandemic [31] due to individuals’ attitudes regarding the disease based on their religious predisposition. Although knowledge score may be high, attitude towards the disease can also be significantly affected by perception [19].

The World Health Organization has stipulated guidelines and safety practices to prevent and mitigate SARS-CoV-2 transmission and progression, including handwashing and wearing face masks [32]. From this survey, most of the respondents seem conversant with these, as was evident in their perception of prioritizing safety practices. Additionally, traditional medicine is gaining traction and presumed to be a safer alternative to conventional medicine; this could explain the opinion of some of the respondents of this study that they would resort to traditional medicine if they contract the virus. Furthermore, governments have introduced policies and measures such as temporary travelling restrictions, quarantine of international travellers, wearing face masks, and social distancing, to curb the infection; these decisions influence perception of the disease [23]. This can be inferred from the fact that most of our respondents have a level of formal education, which could translate to an understanding of the government intervention strategies, as about one-third of them deem government intervention as ’good’.

The findings of this survey must be interpreted with a few limitations in mind. This online survey may have resulted in a selection bias for those with higher formal education and acumen for internet and social media; thus, limiting the breadth of respondents across the sociodemographic. Also, the nature of the survey might have favoured respondents (both high and low formal education) in the major cities where internet and mobile network coverage is high; thus, alienating people without internet access or those who cannot access the media. However, this will not invalidate the findings of this survey. Finally, this survey is a cross-sectional study and cannot allow any inferences to be made on causality and temporality, concerning perception and the sociodemographic factors.

## Conclusions

Most West Africans have basic knowledge of COVID-19 and a positive attitude, with likely proactive practice towards the disease. However, results showed that these varied across countries and are influenced by the types of settlements. Therefore, the health and education authorities in various countries should tailor public health interventions towards enforcing preventative measures against this pandemic, with a special focus on the most impoverished communities, the rural and the suburban settings. The present survey highlights the need for a more extensive survey that cuts across the socio-demographic divide to properly ascertain the impact of COVID-19 interventions and general knowledge, including in-person questionnaire administration and interviews, for wider coverage.

## Supplementary Information

**Additional file 1. **Survey Questions.

## Data Availability

The dataset used and/or analysed during the current study are available from the corresponding author on a reasonable request.

## Abbreviations

COVID-19: Coronavirus disease 2019
SARS-CoV-2: Severe Acute Respiratory Syndrome Corona Virus 2
URL: Uniform Resource Locator
ECBAS: Ethics Committee for Basic and Applied Sciences
