# Supplementary material for: Knowledge, attitude and perception of West Africans towards COVID-19: a survey to inform public health intervention
Source: BMC Public Health. 2022 Mar 5;22:445. doi: 10.1186/s12889-022-12814-9 (PMC8898084; doi:10.1186/s12889-022-12814-9)
Supplement: Supplementary file 1 — Additional file 1. Survey Questions. [file 12889_2022_12814_MOESM1_ESM.docx]

**Supplementary Material: Survey Questions**

**Knowledge, Attitude and Perception of West Africans towards COVID-19: A Survey to inform Public Health Intervention**

Aniefiok John Udoakang^1,2*^, Alexandra Lindsey Djomkam Zune^1,3^, Kesego Tapela^1,3^, Oloche Owoicho ^1,3,4^, Ifeoluwa Kayode Fagbohun^5^, Claudia Adzo Anyigba^1,3^, Mat Lowe^6^, Nora Nghochuzie Nganyewo^1,2,7^, Bineta Keneme^8^, Frances Ngozi Olisaka^9^, Agatha Nkem Henry-Ajala^10^, Mary Aigbiremo Oboh^7^, Lily Paemka^1,3^, [Lucas N Amenga-Etego](https://journals.sagepub.com/action/doSearch?target=default&ContribAuthorStored=Amenga-Etego%2C+Lucas+N)^1,3^

*^1^ West African Centre for Cell Biology of Infectious Pathogens* *(WACCBIP), College of Basic and Applied Sciences, University of Ghana, Legon, Accra, Ghana*

*^2^Department of Biological Sciences, University of Medical Sciences, Laje Road, P.M.B. 536, Ondo City, Ondo State, Nigeria*

*^3^Department of Biochemistry, Cell and Molecular Biology, College of Basic and Applied Sciences, University of Ghana, Accra, Ghana*

*^4^Department of Biological Sciences, Benue State University, Makurdi, Nigeria*

*^5^Public Health Division, Nigeria Institute of Medical Research*

*^6^Society for the Study of Women´s Health (SSWH), Old Yundum, Gambia*

*^7^Medical Research Council Unit, The Gambia at the London School of Hygiene and Tropical Medicine, Banjul, The Gambia.*

*^8^Equipe Génétique et Gestion pour les Populations, Département de Biologie Animale, Faculté des Sciences et Techniques, Université Cheikh Anta Diop de Dakar, BP. Box 5005 Dakar, Sénégal*

*^9^Environmental and Public Health Microbiology, Department of Biological science, Benson Idahosa University, P.M.B 1100, Benin City, Edo state. Nigeria*

*^10^Department of Zoology, Parasitology & Bioinformatics Unit, University of Lagos, Nigeria*

*Corresponding Author: Aniefiok Udoakang, aniefiokjohn.udoakang@ucad.edu.sn

***Demographic characteristics***

1. **What is your Nationality?** * [Choose from all West African countries].
2. **What is your country of residence during this pandemic?** * [Choose from all African countries and others].
3. **Gender*** [Female/Male].
4. **Age as at last birthday*** [_____].
5. **Marital status*** [Single, Married, Divorced, Widowed].
6. **How would you describe the area in which you live?** *

- City/Town (Urban),
- Town (Suburban),
- Village (Rural)].

1. **How many people are living in your household, including you?** * [_____].
2. **What is your religion?** *

- Christian
- Atheist
- Islam
- Traditional African Religion
- None

1. **What is your highest educational degree?** * [Primary, Junior Secondary, Senior Secondary/High School, Bachelor’s degree, Post graduate, Vocational].
2. **Total number of years of formal education** [_____]
3. **Occupation***

- Academic/Researcher
- Entrepreneur
- Public/Private servants, excluding Academics
- Students
- Unemployed
- Others

***Knowledge based questions***

1. **What is your source of COVID-19 information**? Tick all that applies*

- Internet websites
- Social media (WhatsApp, Twitter, Instagram, Facebook, etc.)
- Media (Television, Newspaper and Radio)
- Family and Friends
- Others

1. **Are you worried that you or a close person may contract the virus**? * [Yes/No]
2. **Have you or anybody that you know contracted COVID-19?** * [Yes/No/I don't know]
3. **How is COVID-19 transmitted?** (Please, tick all applicable) *

- By respiratory droplets when an infected person coughs, sneezes or speaks
- Touching contaminated surfaces and then touching your face
- Eating bush meat or wild animals (like bat)
- Contaminated foodstuffs
- Blood transfusion
- Handshake
- I don't know
- Others

1. **Which of the following actions helps to prevent getting infected with or spreading COVID-19? ***

|  | True | False | I don’t know |
| --- | --- | --- | --- |
| Avoid touching your eyes, nose and mouth with unwashed hands |  |  |  |
| Eating garlic, ginger, taking lemon or drinking neem teem |  |  |  |
| Washing of hands after handshake or touching surfaces |  |  |  |
| Covering your month when coughing or sneezing |  |  |  |
| Avoid close contact with people who are sick |  |  |  |
| Washing your hands after touching money |  |  |  |
| Gargling mouthwash and/or saline water |  |  |  |
| Taking food supplements e.g Vitamin C |  |  |  |
| Use of alcohol-based hand sanitizers |  |  |  |
| Steaming, taking a hot bath or sauna |  |  |  |
| Getting a vaccination against flu |  |  |  |
| Staying under the sun |  |  |  |
| Wearing a face mask |  |  |  |
| Taking antibiotics |  |  |  |

1. **The main clinical symptoms of COVID-19 are chest pain, fever, muscle pain, dry cough, shortness of breath**: * [Yes/No/I don’t know/Others]
2. **Unlike the common cold, stuffy nose, runny nose, and sneezing are less common in persons infected with the COVID-19**. * [Yes/No/I don’t know]
3. **Is there currently a vaccine that protects against COVID-19?** * [Yes/No/I don’t know]
4. **There is currently no effective cure for COVID-19, but early symptomatic and supportive treatment can help most patients recover from the infection**. * [Yes/No/I don’t know]
5. **Not all patients with COVID-19 will develop into severe cases. Only those who are elderly, have chronic illnesses, and are obese are more likely to be severe cases**. * [True/False/I don’t know]
6. **Isolation and treatment of people who are infected with COVID-19 are effective ways to reduce the spread of the virus.** * [Strongly disagree/Disagree/Neutral/Agree/Strongly agree]
7. **COVID-19 is diagnosed by isolating the virus from**.... (Please tick all applicable) *

- Swabs from the back of the nose and throat
- Fluid from the lungs
- Faecal sample
- Sputum
- Blood
- Urine
- All of the above
- I don’t know
- Others

1. **Who are the most at-risk people with COVID-19?** Please, Tick all applicable *

- People with diagnosed chronic medical conditions
- People with compromised immune system
- People in detention facilities e.g. prisons
- People in hostels and boarding facilities
- Elderly people
- Children
- Youths

***Attitude-based questions***

1. **What will you do if you or a relative is diagnosed with COVID-19?***

- Continue with usual activities, it will resolve on its own
- Go to hospital or self-isolate for treatment
- Self-isolation, it will resolve on its own
- Speak with a pharmacist
- Herbal treatment
- Self-medication
- Others

1. **If there is a vaccine for COVID-19, would you accept to be vaccinated?** * [Yes/No/I don’t know]

***Perception questions***

1. **1 being less likely and 5 being most likely, where do you think you run a greater risk of contracting COVID-19?** *

|  | 1 | 2 | 3 | 4 | 5 |
| --- | --- | --- | --- | --- | --- |
| Public gathering such as churches, mosques, cinemas and restaurants |  |  |  |  |  |
| Public transports such as buses, trains, airplanes, etc |  |  |  |  |  |
| Public places such as markets |  |  |  |  |  |
| At home with family/friends |  |  |  |  |  |
| Shops and malls |  |  |  |  |  |
| School or work |  |  |  |  |  |
| Hospitals |  |  |  |  |  |

1. **On a scale of 1 - 5, 1 being very badly, how well do you think that your government is managing the spread of COVID-19?** * [1/2/3/4/5]
2. **Do you think that prompt measures are taken to curb the spread of the disease in your country?** * [Yes/No/I don’t know]
3. **Who should be tested for COVID-19 infection?** (Please tick all applicable) *

- Anyone who lives or works in a high risk setting eg. Health care workers, prisons or other closed settings
- Scientists whose research involves close contact with the virus or infected people
- Overseas travellers
- Everybody
- Others

31. It is natural to be tempted to look up the answer to a question. If you did, for approximately how many of the questions above did you look up the answer online or consult before responding? […..]

*Compulsory questions
